# Ovarian and Breast Cancer Spheres Are Similar in Transcriptomic Features and Sensitive to Fenretinide

**DOI:** 10.1155/2013/510905

**Published:** 2013-10-08

**Authors:** Haiwei Wang, Yuxing Zhang, Yanzhi Du

**Affiliations:** ^1^Key Laboratory of Stem Cell Biology, Institute of Health Sciences, Shanghai Institutes for Biological Sciences (SIBS), Chinese Academy of Sciences (CAS) and Shanghai Jiao Tong University School of Medicine (SJTU-SM), 225 Chong-Qing South Road, Shanghai 200025, China; ^2^Graduate School of the Chinese Academy of Sciences, Beijing 100049, China

## Abstract

Cancer stem cells (CSCs) are resistant to chemotherapy and are ability to regenerate cancer cell populations, thus attracting much attention in cancer research. In this report, we first demonstrated that sphere cells from ovarian cancer cell line A2780 shared many features of CSCs, such as resistance to cisplatin and able to initiate tumors in an efficient manner. Then, we conducted cDNA microarray analysis on spheres from ovarian A2780 cells, and from breast MCF7 and SUM159 cells, and found that molecular pathways underlying spheres from these cancer cell lines were similar to a large extent, suggesting that similar mechanisms are involved in the genesis of CSCs in both ovarian and breast cancer types. In addition, we showed that spheres from these cancer types were highly sensitive to fenretinide, a stimulus of oxidative stress-mediated apoptosis in cancer cells. Thus, our results not only provide important insights into mechanisms underlying CSCs in ovarian and breast cancer, but also lead to the development of more sophisticated protocols of cancer therapy in near future.

## 1. Introduction

Cancer stem cells (CSCs) or tumor-initiating cells (TICs) were first identified in leukemia [1] and lately were found in solid tumors such as breast [2], brain [3], colon [4], pancreatic cancer [5], and ovarian cancers [6]. CSCs shared two important features with normal stem cells including self-renewal and differentiation. CSCs are important for tumor growth and recurrence, thus attracting much attention in cancer researches [7–9]. Although several cell surface markers such as CD133 and CD44 are successfully used to identify CSCs in some tumor types [10], the identification of CSCs in many other types of tumors is still a challenging issue due to the lack of specific markers. Alternatively, the sphere cell culture represents a widely used method to enrich CSCs. This method was firstly used for* in vitro* culture of normal breast and brain stem cells [11] and subsequently used for CSC studies [12].

Epithelial ovarian cancer is an extremely aggressive disease, without early symptoms whereas with rapid progression [13]. Breast cancer and ovarian cancer are different types of cancer, whereas they share many similar features pathologically and therapeutically. For instance, *BRCA1* and *BRCA2 *are known the breast cancer susceptibility genes whereas they are also correlated with high risk of ovarian cancer [14, 15]. Moreover, anticancer drugs commonly used to treat breast cancer, such as tamoxifen, appear to show great clinical intervention in ovarian cancer [16]. Although, significant progress has been made in the treatment of ovarian and breast cancers over the past decades, most of the patients eventually relapse and die from chemotherapy-resistant disease [17]. Lines of evidence indicate that CSCs are the source of the resistance and recurrent in these cancers, and thus better clinical outcomes are likely to be achieved once CSCs are eliminated [7]. 

Fenretinide, a derivative of vitamin A, has shown its antitumor activity in many tumor types, with low cytotoxicity to normal cells and high clinical safety [18, 19]. Relative to other retinoids, fenretinide exerts distinct biologic effects, preferentially engaging in the apoptotic pathways through generation of ROS [20], activation of lipoxygenase, and induction of endoplasmic reticulum (ER) stress [21]. It is suggested that this chemopreventive agent fenretinide might kill cancer cells at the early stage, and recently we have shown that fenretinide preferentially eradicates acute myelogenous leukemia (AML) [38] and chronic myeloid leukemia (CML) stem/progenitor cells [22]. Accordingly, it is of great interest to investigate whether fenretinide could selectively target CSCs in solid tumors.

## 2. Materials and Methods

### 2.1. Cell Lines and Cell Culture

Human ovarian cancer A2780 cells were cultured in RPMI 1640 supplemented with 10% fetal bovine serum (PAA, Linz, Austria). Human breast cancer cell lines MCF7 and SUM159 were cultured in DMEM (Invitrogen, Carlsbad, CA, USA) supplemented with 10% fetal bovine serum. A2780, MCF7, and SUM159 cells were cultured to form the spheres in serum-free media. The sphere formation media (SFM) were DMEM-F12 (Invitrogen) supplemented with 2% B-27, 20 ng/mL epidermal growth factor (Sigma-Aldrich St. Louis, MO, USA), 5 *μ*g/mL insulin (sigma), and 0.4% bovine serum albumin (Amresco Inc., Cleveland, OH). Dissociated cells were seeded in SFM with or without fenretinide treatment and the spheres were observed and photographed using inverted microscope. All the cells were cultured at 37°C in a humidified atmosphere with 5% CO_2_.

### 2.2. MTT Cell Viability Assay

For the cell viability assay, cells were seeded in 24-well plates overnight, and then cells were treated with indicated agents for indicated time course. 100 *μ*L MTT solutions (5 mg/mL in PBS) were added to each well for an additional 3 hrs at 37°C. The MTT was dissolved with 1 mL dimethyl sulfoxide for 1 hr and the absorbance was determined and recorded with a spectra microplate reader DU800 (Beckman Coulter, Brea, CA, USA). For the sphere cell viability, the spheres were formed in sphere-forming conditions and enzymatically dissociated cells were cultured in the suspension plate, and then cells were treated with indicated agents. 

### 2.3. Cell Apoptosis Assay

Cell apoptosis was detected using a FITC-Annexin V Apoptosis Detection Kit (BD Biosciences Pharmingen, San Diego, CA, USA). Briefly, cells were seeded in 6-well plates and exposed to treatments. The floating and trypsinized adherent cells were then collected and detected by flow cytometry. For the apoptosis of sphere cells, the sphere cells were first formed in the SFM, then trypsinized, and seeded in the suspension dish. After treatment with cisplatin (sigma) and fenretinide (sigma), cells were then collected and detected. The total percentage of Annexin V+ PI− and Annexin V+ PI+, as indicated the apoptotic cells, was quantified.

### 2.4. ROS Detection

The intracellular ROS was detected by 2′,7′-dichlorofluorescein diacetate (DCFDA, Sigma). After seeded in 6-well plates for 24 hrs, the cells were pretreated with 100 *μ*M vitamin C and then treated with indicated agents. Cells were collected and cultured with DCFDA for 30 mins. The florescence intensity was detected by flow cytometry. Untreated cells were used as normalization control.

### 2.5. Subcutaneous Model of Ovarian Tumorigenesis

The animal experiments were approved by the Committee on Laboratory Animal Research of Shanghai Jiaotong University, China, and conducted according to the guidelines of the Laboratory Animal Center of Shanghai Jiaotong University School of Medicine. Six-to-eight-week-old female NOD-SCID mice were purchased from Shanghai Slac Animal Center (Shanghai, China). 10000 A2780 sphere cells were injected subcutaneously into the left inguinal area of the mice and the same number of parental cells to the right inguinal area of the same mice. The tumor growth was monitored every five days. After 40 days, the mice were sacrificed and the tumors were excised from the body for analysis.

### 2.6. Reverse Transcription-PCR (RT-PCR) Analysis

Cellular RNA was isolated by Tri-Reagent (Molecular Research Center) using the manufacturer's instructions, DNA was removed from the samples using DNase treatment (DNA-free kit, Ambion Applied Biosystems), and cDNA was synthesized from the purified RNA using Moloney murine leukemia virus reverse transcription kit (Promega, Madison, WI, USA). GAPDH primer sets were used to produce a normalization control. Real-time RT-PCR was carried out in triplicate with the SYBR Green PCR Master Mix (Applied Biosystems, CA, USA) and a 7900HT Fast Real-Time PCR machine (Applied Biosystems).

### 2.7. Microarray Hybridization and Data Mining

Total RNA from A2780, MCF7, and SUM159 parental and sphere cells were amplified and labeled with biotin according to the standard Affymetrix protocol. The fragmented, biotinylated cDNA was then subjected to hybridization with the GeneChip Human Genome-U133 Plus 2.0 array (Affymetrix, Santa Clara, CA). DAVID Bioinformatics Resources analyses of biological themes were performed to explore the underlying themes of those statistically significant differentially expressed genes in terms of biological relevance, for example, functional relevance as revealed by Gene Ontology (GO) enrichment analysis [23] and regulatory relevance as revealed by UCSC conserved transcription factor binding site (TFBS) enrichment analysis [24]. The Benjamini-Hochberg-derived step-up procedure of False Discovery Rate (FDR) was applied to account for multiple hypothesis testing, thus to assess the significance of the biological theme enrichments. The transcriptome profilings of A2780, MCF7, and SUM159 parental and sphere cells are available at GEO accession GSE43657.

### 2.8. Statistical Analysis

Statistical analysis was performed using Student's *t*-test. A *P* value less than or equal to 0.05 was chosen to be statistically significant difference. 

## 3. Results 

### 3.1. Sphere Cells from Ovarian Cancer Cell Line A2780 Are Cisplatin-Resistant

**Figure 1:**
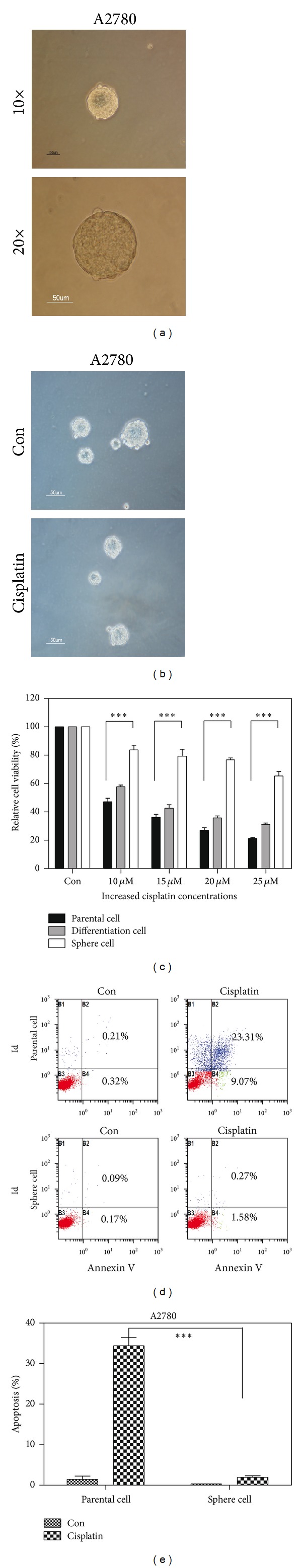
Sphere cells from ovarian cancer cell line A2780 were cisplatin resistant. (a) The sphere was from a single A2780 cell when A2780 cell was cultured in sphere-forming conditions. The sphere was photographed using inverted microscope after the cell was seeded on 96-well suspension culture plates for 5 days. (b) A2780 cells were seeded in sphere-forming condition without (up) or with (down) 20 *μ*M cisplatin. Five days later, the spheres were photographed using inverted microscope. (c) The ovarian cancer A2780 cells (parental cell for short) and enriched sphere-forming cells (sphere cell) were seeded in plates and treated with different concentrations of cisplatin for 48 hrs. Sphere cells reseeded with full serum media for 3 days (differentiation cell) were also treated with different concentrations of cisplatin for 48 hrs. Cell viability was determined by MTT assay. ∗∗∗, statistically significant difference between A2780 unenriched cells and enriched sphere cells treated with cisplatin (*P* < 0.001). (d) A2780 parental and sphere cell were treated with 20 *μ*M cisplatin; after 48 hrs, the apoptotic cells were detected through Annexin V/PI assay. (e) Means and standard errors of total number of apoptotic cells from three experiments in Figure 1(d) were shown. ∗∗∗, statistically significant difference between A2780 unenriched cells and enriched sphere cells treated with cisplatin (*P* = 0.0002). Data are representative of values from three independent experiments.

Under a serum-free culture condition, normal stem cells and CSCs can form spheres, which are usually used for the expansion of stem cells *in vitro* [12]. To ensure that sphere cells were single-clone derived, we conducted a limited-dilution of A2780 cells in 96-well plates. After 5 days in culture, A2780-originated spheres were observable under a conventional light microscope (Figure 1(a)). Cisplatin is one of the firstline agents in chemotherapy of ovarian cancer [25]. To test whether sphere cells of this setting were resistant to cisplatin, we compared sphere formations in culture plates with and without the presence of cisplatin. As shown in Figure 1(b), the impact of cisplatin on the sphere formation was minor, even if a high concentration (20 *μ*M) of the agent was used in the culture. Similarly, when the impact of cisplatin on cell viability was examined, respectively, in the parental A2780 cells, the differentiated sphere cells, and the sphere cells (Figure 1(c)), significant difference (*P* < 0.001) was detected between the sphere cells and the A2780 cells/the differentiated sphere cells. In addition, we conducted cell apoptosis assays in the A2780 cells and the sphere cells, with or without the presence of cisplatin. As shown in Figures 1(d) and 1(e), a prominent induction of apoptosis was only observed in the A2780 cells treated with cisplatin, not in the sphere cells treated with the agent. Taken together, these results indicate that the sphere cells of this setting may mimic CSCs of ovarian cells, resistant to the conventional chemoagent cisplatin.

### 3.2. Sphere Cells from Ovarian Cancer Cell Line A2780 Were Highly Tumorigenic

**Figure 2:**
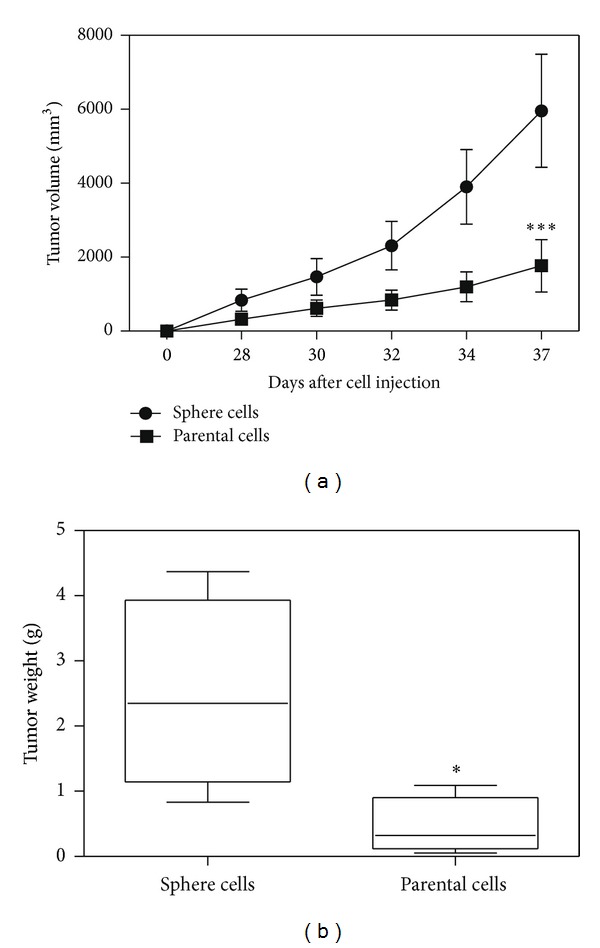
Sphere cells from the ovarian cancer A2780 cell line were highly tumorigenic. (a) Tumor volume of injected mice was measured at indicated time point after the injection of 10000 sphere cells and the same number of parental cells. Means and standard errors of four mice were shown (*n* = 4). ∗∗∗, statistically significant difference in mean tumor size between the mice injected with enriched sphere cells and mice injected with the same number of unenriched cells (*P* < 0.001). (b) Means and standard errors of tumors weight from four mice in each group were measured. ∗, statistically significant difference in mean tumor weight between two groups (*P* < 0.05).

In addition to treatment resistance, CSCs are considered to be drivers of tumor progression. Accordingly, an equal number of the parental or sphere cells (i.e., approximately 10,000) were injected into inguinal area of NOD-SCID mice. Indeed, significantly increased initiation and growth of tumors were observed in mice injected with the sphere cells (Figure 2(a)). Similarly, the median of tumor weights in mice injected with the sphere cells was significantly greater than that in mice injected with the parental cells (Figure 2(b)). These results appear to be consistent with the notion that CSCs drive tumor progression. 

### 3.3. Implications of Molecular Pathways Underlying the Sphere Cells through Transcriptomic Analysis

**Figure 3:**
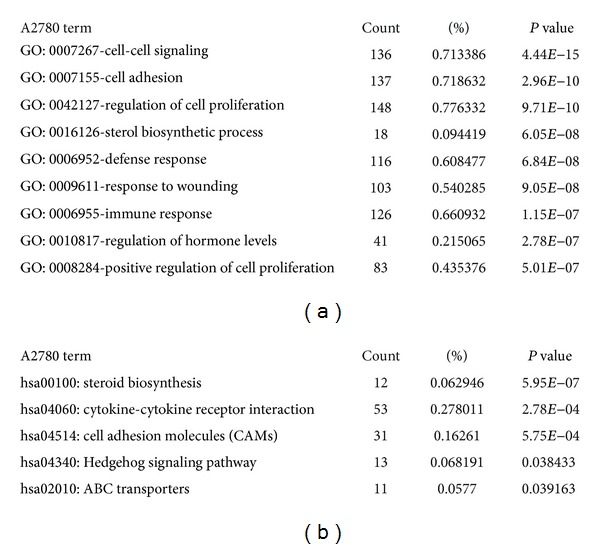
The CSCs molecular signature by GO and pathway analysis in ovarian cancer cells. RNA was extracted from A2780 spheres cells and their corresponding parental cells, and then profiled to Affymetrix microarray system. The regulated genes were selected based on 2-fold change threshold. The GO (a) and pathway (b) analysis of the regulated genes were enriched through DAVID Bioinformatics Resources. The most enriched functions were shown.

Similar to a previous approach [26], we applied cDNA microarray to identify transcriptomic features associated with the sphere cells. Accordingly, total mRNA from A2780 parental or sphere cells was extracted and profiled using a whole genome array (Affymetrix Human Genome-U133 Plus 2.0). After data normalization and comparison, a total of 2,812 genes were found characteristically associated with the sphere cells. These genes were then loaded to a database (DAVID Bioinformatics Resources) [27] for the recognition of biological processes potentially important for the sphere cells. As illustrated in Figure 3(a), significant processes associated with the sphere cells were highlighted by cell-cell signaling, cell adhesion, regulation of cell proliferation, sterol biosynthesis, defense response, response to wounding, and hormone regulation. Accordantly, molecular pathways, as significantly revealed through KEGG_analysis, were suggested by steroid biosynthesis, cytokine-cytokine receptor interaction, cell adhesion molecules, Hedgehog (Hh) signaling pathway, and ABC transporters (Figure 3(b)). Of note, the Hh signaling is known to be essential for stem cell self-renewal, organ homeostasis, and wound repair in many tissues, and constitutively activated in many types of cancer. Thus, targeting this signaling pathway has shown promising potential in cancer therapy [27, 28]. The association of the Hh signaling pathway with the sphere cells implicates an important role played by this signaling in the genesis of CSCs in ovarian cancers. Another interesting pathway associated with the sphere cells was represented by ABC transporters. It is reported that CSCs express high levels of ATP-binding cassette (ABC) transporters [29], such as ABCB5 [30] and the half-transporter ABCG2 [31], which may protect CSCs from apoptosis induced by chemotherapeutic agents. The association of this pathway with the sphere cells may therefore explicate why CSCs of ovarian cancer are resistant to cisplatin.

### 3.4. Identification of Common CSC Pathways in Ovarian and Breast Cancer Cells

**Figure 4:**
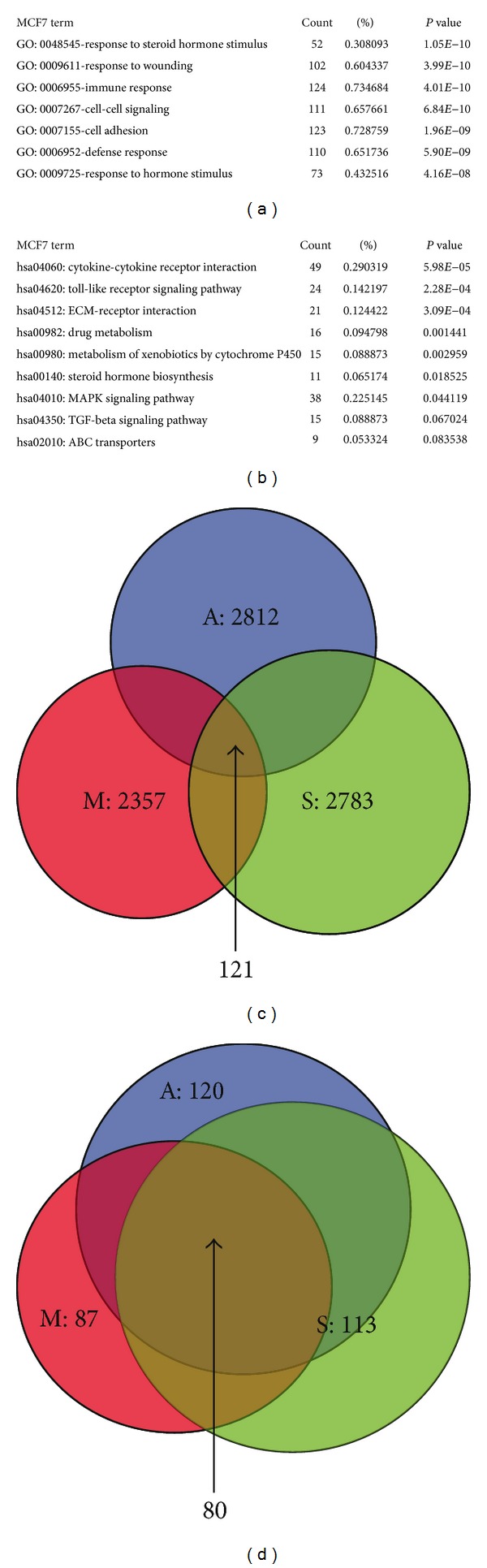
Identification of CSCs signatures in ovarian and breast cancer cells. Similar procedures were involved to identify changed genes in MCF7 and SUM159 sphere cells. GO (a) and pathways (b) of the changed genes in MCF7 spheres were enriched through DAVID Bioinformatics Resources; the most enriched functions were shown. (c) The number of changed genes was shown. A: ovarian cancer A2780 cells, M: breast cancer MCF7 cells, and S: breast cancer SUM159 cells. (d) The number of enriched transcription factors from the changed genes in A2780, MCF7, and SUM159 spheres cells was analyzed through DAVID Bioinformatics Resources.

Steroid biosynthesis and hormone regulation appear to be characteristic features associated with the ovarian cancer-derived sphere cells. It is thus deducible that such features might be associated with sphere cells derived from breast cancer as well. Accordingly, sphere cells were prepared, respectively, from breast cancer MCF7 and SUM159 cells, as described previously [32], and applied to the whole-genome cDNA microarray, as mentioned previously. The number of genes associated with the MCF7 spheres appeared to be 2,357 and that associated with the SUM159 spheres were 2,783. Accordingly, GO in those sphere cells were suggested by cell-cell signaling, cell adhesion, sterol biosynthetic process, defense response, and regulation, of hormone level in MCF7 sphere (Figure 4 and SUM159 data not shown). The pathways were noted to steroid biosynthesis, cytokine-cytokine receptor interaction and cell adhesion molecules (Figure 4(b) and SUM159 data not shown). But the drug metabolism (*P* < 0.01) and metabolism of xenobiotics by cytochrome P450 (*P* < 0.01) pathways [33] suggested that MCF7 CSCs used such mechanisms for drug resistance rather than ABC transporters (*P* > 0.05).

Genes associated with sphere cells may be regulated by specific transcription factors. Totally, 120 transcription factors were enriched in A2780 cells, 87 in MCF7 cells, and 113 in SUM159 cells. To our surprise, although only nearly 5% (121) genes were common in A2780, MCF7, and SUM159 spheres (Figure 4(c)), most of enriched transcription factors were the same (Figure 4(d)). Genes and transcription factors were in supplementary data (See supplementary Table S1, Table S2, Table S3, and Table S4 available online at http://dx.doi.org/10.1155/2013/510905). Among those transcription factors, STAT5 had been reported to regulate the self-renewal of leukemia stem cell [34]. STAT3 mediated the multidrug efflux in breast and ovarian tumor cells, thus contributing to the resistance of CSCs [35]. Transcription factors NF1 [36] and AP1 [37] also committed important roles in the regulation of progenitor cells in tumor initiation. The common enriched GO (Figures 3(a) and 4(a)) and pathways (Figures 3(b) and 4(b)) implied that although the different genes were expressed in ovarian and breast cancer CSCs, the involved biological processes and pathways were similar.

### 3.5. Fenretinide Preferentially Targeted on Sphere Cells

Previously, our lab has shown that fenretinide could preferentially eradicate AML and CML stem cells [22, 38]. Here, we tested whether fenretinide could selectively target on CSCs in ovarian and breast cancer.

**Figure 5:**
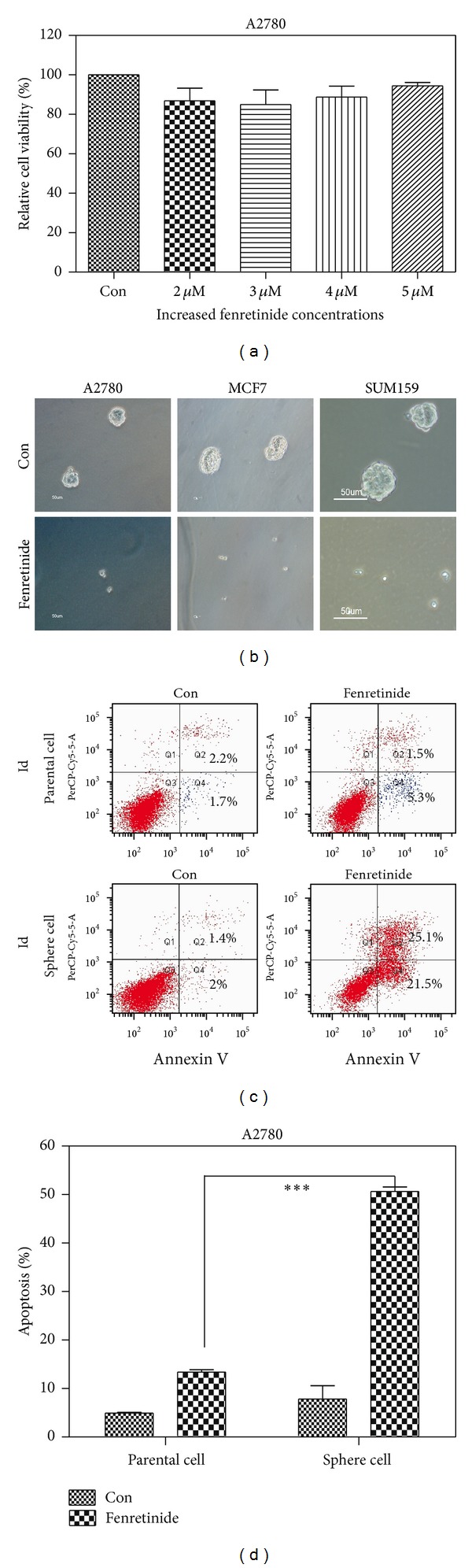
Fenretinide inhibited the formation of sphere cells in ovarian and breast cancer cells. (a) Data summary and analysis of cell viability in A2780 cells. A2780 cells were treated with different concentrations of fenretinide for 48 hrs. Cell viability was determined by the MTT assay. Means and standard errors of three independent experiments were shown. (b) Illustration showed the A2780, MCF7 and SUM159 sphere cells. A2780, MCF7, and SUM159 cells were seeded in sphere-forming condition with (down) and without (up) 3 *μ*M fenretinide, the spheres were photographed using inverted microscope (magnification 10x). The results were obtained from three independent experiments. (c) Representative dot-plots illustrating apoptotic status in A2780 parental and sphere cells. A2780 parental and sphere cells were treated with 3 *μ*M fenretinide for 48 hrs. The apoptotic cells were detected through Annexin V/PI assay. (d) Data summary and analysis of apoptotic index in A2780 parental and sphere cells. Means and standard errors of apoptosis percentages of A2780 parental and sphere cells treated with fenretinide from three experiments were shown. ∗∗∗, statistically significant difference between A2780 parental and sphere cells treated with fenretinide (*P* < 0.001).

A2780 parental cells were relatively not sensitive to fenretinide (Figure 5(a)). But when A2780 sphere cells were cultured with fenretinide in serum-free media, the fenretinide treatment group detected few numbers of spheres formation (Figure 5(b)). Fenretinide had been used in breast cancer treatment [39]. It is of great interest to investigate whether fenretinide could also eradicate the breast CSCs. As Figure 5(b) showed, the formation of breast spheres in MCF7 and SUM159 cells was inhibited. 

The different effects of fenretinide in the A2780 parental and sphere cells were quantified in cell apoptosis assay. As shown in Figures 5(c) and 5(d), apoptotic cells were barely detected in the parental cells, but in the sphere cells, more than 40% of apoptotic cells were detected (*P* < 0.0001). These results suggested that fenretinide may represent a promising candidate to eradicate cancer primitive/stem cells.

### 3.6. Fenretinide Involved ROS Induction, ER Stress, and Cell Cycle Progression in A2780 Cells

**Figure 6:**
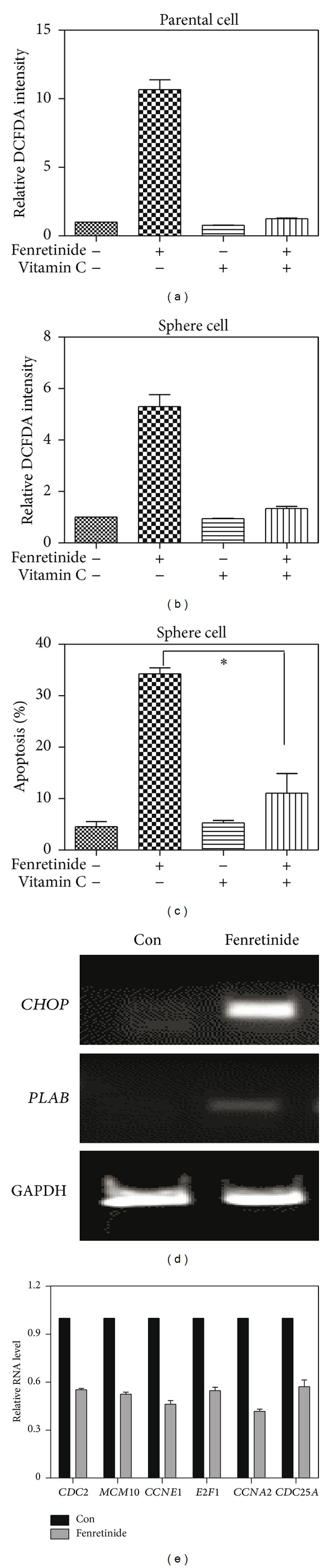
Fenretinide induced ROS induction, ER stress, and cell cycle progression arrest in A2780 cells. (a) Data summary and analysis of DCFDA intensity in A2780 parental cells. DCFDA intensity was detected after 3 *μ*M fenretinide or 100 *μ*M vitamin C or combined fenretinide and vitamin C treatment for 3 hrs in A2780 parental cells. The untreated parental cells were used as normalization control. Means and standard errors of relative ROS level from three experiments were shown. (b) Data summary and analysis of DCFDA intensity in A2780 sphere cells. DCFDA intensity was detected after fenretinide or vitamin C or combined fenretinide and vitamin C treatment for 3 hrs in A2780 enriched sphere cells. (c) Data summary and analysis of apoptosis in A2780 enriched sphere cells. Apoptotic cells were detected through AnnexinV/PI assay, after fenretinide or vitamin C or combined fenretinide and vitamin C treatment for 24 hrs in A2780 enriched sphere cells. Means and standard errors of apoptosis percentages from three experiments were shown. ∗, statistically significant difference between A2780 enriched sphere cells treated with fenretinide combined vitamin C treatment and fenretinide treatment group (*P* = 0.0278). (d) mRNA expression of *CHOP* and *PLAB* genes in A2780 sphere cells. A2780 sphere cells were treated with 3 *μ*M fenretinide for 6 hrs. mRNA expression of *CHOP* and *PLAB* genes were detected by PCR. Products were separated by electrophoresis, *GAPDH* as a housekeeping gene for loading control. (e) Data summary and analysis of mRNA expression in A2780 sphere cells. mRNA expression of *CDC2*, *MCM10*, *CCNE1*, *E2F1*, *CCNA2,* and *CDC25A* was detected by real-time RT-PCR. The results were obtained from three independent experiments.

ROS balance between generation and elimination is important for normal cell functions. Cancer cells produce more ROS than normal cells [40]. Malignant cells would be more dependent on an antioxidant system for cell survival and be more vulnerable to oxidative insults. Fenretinide was known as an ROS inducer [41]. In many tumor cell lines, fenretinide-induced apoptosis was largely mediated by oxidative stress [42]. However, the involved mechanisms likely depended on tumor types [43]. So, we studied whether the mechanisms on fenretinide eliminating CSCs were related to ROS generation or not. The intracellular ROS was detected by DCFDA intensity. Fenretinide inducted nearly tenfold increased ROS level in A2780 parental cells (Figure 6(a)) and fivefold induction in sphere cells (Figure 6(b)). And the induction of ROS in parental and sphere cells could be eliminated by pretreatment with vitamin C (Figures 6(a) and 6(b)). More importantly, accompanied with the elimination of the induction of ROS by vitamin C, the induction of apoptosis by fenretinide was also reversed (Figure 6(c), *P* = 0.0278). Also, *CHOP* and *PLAB* genes, the markers of ER stress, were up-regulated (Figure 6(d)). The results suggested that induction ROS and ER stresses were participated in fenretinide-related functions in sphere cells.

Fenretinide inhibited the sphere cells proliferation and genes involved cell cycle were analyzed through Real-time PCR assay [21]. Comparing to the untreated cells, genes such as *CDC2*, *MCM10*, *CCNE1*, *E2F1*, *CCNA2,* and *CDC25A* were down regulated after fenretinide treatment (Figure 6(e)). Those genes were essential for the cell cycle control, and those down-regulated genes inhibited the sphere cells proliferation. Yet, those genes also played important regulatory roles in CSCs. E2F1 regulated the stem-like properties of lung cancer [44]. *CDC25A* enhanced proliferation rate and promoted a more undifferentiated cell phenotype, which was similar to stem cells state in neuroblastoma [45]. Decreased expression of those genes not only inhibited cell cycle progression, but also changed the stem state of the sphere cells.

Fenretinide was a multitarget drug. Except for induction of apoptosis through ROS generation, activation of nuclear retinoid receptors and triggering of the mitochondrial caspase cascades were also observed in different cancers. Previously, using high-throughput microarray platform, our lab had studied the complex mechanisms of fenretinide in leukemia cells [22, 38]. In the further studies, we will profile the globe transcriptional signatures of fenretinide treatment and provide deep understandings of the mechanisms of fenretinide in solid tumors.

## 4. Discussion

The cancer stem cell theory suggests that a small population of progenitor cells with extensive self-renewal property determines the tumor initiation, maintenance, progression, and recurrence. Because of the complexity, rarity, and difficulty in getting pure CSC population, the behavior of the cancer stem cells is not clear. And different types of CSCs may use different genes to maintain their properties. Here, based on their renewal characteristics, spheres were used for the expansion of stem cells *in vitro*. In this study, we showed that those sphere cells were resistant to chemotherapy drugs like cisplatin (Figure 1) and more tumorigenic when inoculated into the immune-deficient mouse (Figure 2). Those results suggested that the sphere cells shared the properties of CSCs and could be used as a model to study the functional genes of CSCs and screening drugs to eliminate CSCs *in vitro*.

To study the regulation of CSCs, we identified the associated genes in ovarian cancer A2780 and breast cancer MCF7 and SUM159 sphere cells using microarray system. Biological processes, pathways, and transcription factors were highlighted. Among them, some pathways like Hedgehog signaling pathway and ABC transporters (Figure 3) and transcription factors like STAT5, STAT3, NF1, and AP1 had conferred the regulation of CSCs, and others may require our further studies. Ovarian and breast cancer, were two kinds of cancer involved in the abnormal regulation of hormone levels. Our data suggested that hormone may play important roles in maintaining CSCs properties. It was also interesting to see that ovarian cancer and breast cancer may use different genes to regulate CSCs, but those genes converged to the same pathways and transcription factors (Figure 4).

The aims of our studies were to investigate the underlying mechanisms of CSCs, thus providing strategies to eliminate those cells and achieving better therapies on cancer patients. Here, we showed that fenretinide, a derivative of vitamin A, could preferentially target on CSCs through induction of ROS and ER stress and inhibition of cell-cycle-related genes. ROS played an important role in the regulation of CSCs and conferred the resistance to radiotherapy [8]. The low levels of the ROS in CSCs made it hard to be eliminated by traditional radiotherapy. Fenretinide was an ROS inducer, inducted ROS both in parental and sphere cells (Figure 6). And the increased level of ROS was higher in the cell line (Figure 6(a) secondary lane tenfold induction compared to Figure 6(b) secondary lane fivefold induction). Yet, comparing to the parental cells, the sphere cells were more sensitive to the fenretinide treatment (Figure 5). So, low levels of ROS in CSCs may present disadvantage to the radiotherapy but give us an opportunity to preferentially target on CSCs cells by carefully designing the ROS inducer drug. ER stress generation and inhibition of cell cycle were also involved in the function of fenretinide. In the further studies, profiling the globe transcriptional signatures of fenretinide treatment may provide deep understandings of how fenretinide works in solid tumor.

Above all, those studies provided an *in vitro* model for the researches of CSCs. Using this model, we identified the regulated genes in CSCs. And the nature of fenretinide targeting these CSCs from ovarian and breast cancer cells may provide the new application in the cancer therapy in near future.

## Supplementary Material

Total RNA from A2780, MCF7, and SUM159 parental and sphere cells was amplified and labeled with biotin according to the standard Affymetrix® protocol. The fragmented, biotinylated cDNA was then subjected to hybridization with the GeneChip® Human Genome-U133 Plus 2.0 array (Affymetrix, Santa Clara, CA). DAVID Bioinformatics Resources analyses of biological themes were performed to explore the underlying themes of those statistically significant differentially expressed genes in terms of biological relevance, e.g., functional relevance as revealed by Gene Ontology (GO) enrichment analysis and regulatory relevance as revealed by UCSC conserved transcription factor binding site (TFBS) enrichment analysis. The Benjamini-Hochberg derived step-up procedure of False Discovery Rate (FDR) was applied to account for multiple hypothesis testing, thus to assess the significance of the biological theme enrichments. The transcriptome profilings of A2780, MCF7, and SUM159 parental and sphere cells are available at GEO accession GSE43657.
Table S1.The common regulated genes in A2780, MCF7 and SUM159 spheres cells.
Table S2. The enriched transcription factors from the changed genes in A2780 spheres cells.
Table S3. The enriched transcription factors from the changed genes in MCF7 spheres cells.
Table S4. The enriched transcription factors from the changed genes in SUM159 spheres cells.
